# Psychological risk factors and cardiovascular disease

**DOI:** 10.3389/fpsyg.2024.1419731

**Published:** 2024-09-30

**Authors:** Valeria Carola, Cristina Vincenzo, Giulia Di Vincenzo, Chiara Morale, Valentina Cecchi, Giampaolo Nicolais

**Affiliations:** Department of Dynamic and Clinical Psychology, and Health Studies, Sapienza University of Rome, Rome, Italy

**Keywords:** cardiovascular diseases, adverse childhood experiences, anxiety, depression, stress coping strategies

## Abstract

**Objectives:**

Cardiovascular diseases (CVDs) are a leading cause of death worldwide, emerging from a combination of several factors. The aim of this review is to define the psychological factors that are significant in the development and progression of these disorders.

**Methods:**

Studies published through 2023 concerning adults with psychological vulnerability factors and/or cardiovascular disease were selected through searches of PubMed, PsychINFO, Science Direct, and Google Scholar.

**Results:**

Psychological stress may influence CVD, in combination with other risk factors, or it can act independently, as in cases of workplace stress, post-traumatic stress disorder, Takotsubo syndrome and bereavement. Coping strategies, anxiety and depression have also been identified as relevant psychological factors in cardiac patients. Adverse childhood experiences are linked to a reduced quality of life and have been identified as significant risk factors for the development of acquired CVDs.

**Conclusion:**

This review demonstrates that several psychological factors affect cardiovascular function. An in-depth study of the psychological correlates of CVDs would allow healthcare professionals to design more effective prevention and intervention programs.

## 1 Introduction

Cardiovascular disease (CVD), including coronary heart disease (CHD), cardiac arrhythmias, stroke, and heart failure, is a leading cause of death worldwide (Correa-Rodríguez et al., 2020). Of these conditions, CHD, presenting as angina or myocardial infarction (MI), is the most common (Roth et al., 2017; Khan et al., 2020).

According to estimates by the World Health Organization (2022), cardiovascular system disorders claim nearly 18 million lives each year, accounting for over 30% of global deaths. Further, their risk of occurrence is governed significantly by such behavioral factors as tobacco use, excessive alcohol consumption, an unhealthy diet and inadequate physical activity. This risk also traces back to physiological factors, including high blood pressure and high blood cholesterol and glucose levels, which are linked to underlying social factors, including aging, income and urbanization.

CVDs manifest progressively as multifactorial disorders that emerge from the competition of genetic, environmental, organic and behavioral factors, prompting clinicians to orient themselves increasingly toward a holistic understanding of the CVD patient. Defining the psychological factors that appear to have a significant role in the development of a cardiovascular disorder, in its progression and in the rehabilitation of such a patient would be essential to better intervene in this clinical context.

An analysis of the literature indicates that studies have generally focused on the in-depth examination of various psychological constructs that are linked to cardiovascular health, including psychosocial stress, stress coping strategies, early-life stress/adverse childhood experiences (ACEs), the attachment system, anxiety and depression. The level of stress in coronary patients appears to be higher than in the general population (Hallman et al., 2003). Prospective epidemiological studies show that chronic stress, in particular, is associated with an excess risk of CHD and a poor cardiovascular prognosis, supporting the involvement of stress as a causal risk factor for CHD (Wirtz and von Känel, 2017). Stressful experiences can significantly affect the development of CVD due to their impact on the body. Specific brain areas, such as those that control the autonomic and physiological response to stress, are crucial in regulating stress-induced cardiovascular reactions.

Moreover, prolonged and intense stress reactions can lead to various cardiovascular risks (Ginty et al., 2017). Among the chronic psychological stress conditions that are most frequently associated with CVD, work stress, social isolation, ACEs, post-traumatic stress disorder (PTSD), bereavement (Carey et al., 2014) and Takotsubo syndrome (TTS) are the most extensively examined (Sharkey et al., 2011). Also, acute emotional stress, such as trauma feelings and intense negative emotions, can trigger acute CHD events in vulnerable patients (Ginty et al., 2017).

Personality traits May play an important role in cardiovascular health. Type D personality, a joint tendency toward negative affectivity (NA) and social inhibition (SI), is related to poor cardiac prognosis (Denollet et al., 2006) and patients with this trait simultaneously tend to experience distress and inhibit the expression of emotions (De Fazio et al., 2012).

Stress coping strategies have been a major focus in health psychology (Lazarus and Folkman, 1984). In the literature, coping strategies have been evaluated as relevant psychological factors in patients with coronary involvement. As part of one’s character, coping strategies consist of behavioral and cognitive efforts that develop to manage stressful encounters (Lazarus and Folkman, 1984). Individuals attempt to use emotion-focused or problem-focused strategies to modify the situation or regulate their emotions. It appears that adaptive coping strategies are protective against CVD events, whereas maladaptive coping strategies pose the same threat as risk factors (Roohafza et al., 2022). Psychopathological conditions that are related to anxiety and depression have been associated with avoidant coping strategies (Finset and Andersson, 2000; Sinyor et al., 1986) and tendency to rationalization (Gillespie, 1997).

Research has also shown that exposure to chronic stress during childhood heightens one’s susceptibility to the development of CVD in adulthood (Rinnewitz et al., 2018). This phenomenon is explained in part by the impact of chronic stress on a child’s cardiovascular system, altering its function permanently and increasing its reactivity; concurrently, this association is mediated by the greater frequency of cardiovascular health risk behaviors, such as substance abuse, sedentary lifestyle and obesity, in individuals who have suffered from ACEs (Nelson et al., 2020; Finkelhor, 2020).

It has been widely demonstrated that early-life stress can derive from the relationships that a child has with caregivers. In particular, these primary relationships are characterized by the experience of attachment—the innate human tendency to form strong emotional bonds with important individuals, primarily to manage emotions when facing potential threats (Bowlby, 1958).

The attachment system is critical in regulating emotions, especially during times of perceived danger, such as when dealing with a severe medical condition. Attachment appears to have a significant influence on behaviors that are related to adherence to medical treatment. Several reports have shown that individuals who develop an insecure attachment style are at risk for chronic health conditions and CVD (Pietromonaco et al., 2013; Robles and Kane, 2014; Meredith and Strong, 2019; McWilliams and Bailey, 2010; Heenan et al., 2020).

The impact of anxious and depressive symptoms on cardiovascular patients has also garnered substantial attention in the last 50 years. Depressive symptoms have been estimated to be prevalent in 31.3% of cardiovascular patients, whereas anxiety symptoms have been reported in 32.9% of cases (Karami et al., 2023). Women are more frequently affected by anxiety and depression (World Health Organization, 2024). Despite the high heterogeneity in the methods for measuring such rates in the literature, causing wide variations in their prevalence and effects, there is agreement that anxiety and depressive symptoms are important risk factors in the clinical course of cardiovascular patients (Karami et al., 2023).

These factors also influence post-operative recovery in cardiac surgery patients (Oxlad et al., 2006; Kidd et al., 2014). In particular, length of stay is influenced by attachment anxiety (Kidd et al., 2014) and PTSD (Oxlad et al., 2006) and has been implicated in a vicious circle with depressive symptomatology, worsening the psychopathological condition and in turn being exacerbated by it (AbuRuz, 2019). Thus, it is clear that these factors contribute to the onset and clinical course of cardiovascular disorders, the study of which by the treatment team can improve the diagnosis and management of patients. No work has analyzed and integrated the available data for these psychological risk factors in CVDs.

Given the significant impact of psychological factors on cardiovascular disease (CVD), this review aims to exhaustively explore the existing scientific literature. By investigating the complex interaction between psychological states and CVD, we seek to promote a greater understanding of these disorders among healthcare professionals involved in patient care. This paper considers CVD from its onset and progression to the critical stages of rehabilitation. Our interest focuses on how the psychological factors considered May contribute to the onset, progression and outcomes of CVD. Specifically, we refer to anxiety, depression, coping strategies, stress in early life and adulthood and its psychobiological correlates. We therefore consider the role of these factors in the various stages of the disease, including risk factor assessment, diagnosis and clinical course. Starting from the available scientific evidences, we thus aspire to offer useful insights into the potential benefits of integrating psychological interventions into the comprehensive care of patients with CVD.

## 2 Methods

A systematic search was conducted in PsychInfo, PubMed, Google Scholar, and Science Direct for studies on psychological variables associated with cardiovascular disorders. Reviews, systematic reviews, longitudinal studies, meta-analyzes, and experimental studies were considered.

The screening process involved studies that included psychological factors associated with cardiovascular risk and/or disease. Specifically, studies were included that considered psychological vulnerability factors searched through the keywords: “anxiety,” “depression,” “early stress” and “adult stress,” “coping strategies,” “attachment,” “ACEs,” “child maltreatment,” “bereavement,” and “length of stay.” In addition, studies involving Takotsubo syndrome and cardiovascular diseases more in general were included. The studies included both clinical patients and volunteer subjects. Pre-clinical and pediatric studies were excluded. The databases search was conducted by four investigators during the period between April 2023 and November 2023.

## 3 Results

### 3.1 Psychological stress and CVD

Stress levels in coronary patients are higher than in the general population (Mustață, 2021; Table 1). Psychological stress, defined as any mental tension or physiological response to a stimulus, May be involved in CVD conditions, in addition to hypertension, diabetes, obesity and a family history of CVD, and might also independently have a significant relationship with CVD events (Sadr Bafghi et al., 2018; Ginty et al., 2017). Stress accounts for approximately 30% of the attributable risk of acute MI and is often associated with self-destructive behaviors and medication non-compliance (Das and O'Keefe, 2006). Further, MI has been linked to a higher prevalence of various types of stress, particularly in the period prior to the cardiac event (Rosengren et al., 2004).

**Table 1:** Selected studies describing the implication of stress, coping strategies and personality traits in CVDs.

Authors	Country	Study design	Population	Methods	Outcomes
Wirtz and von Känel (2017)	Germany and Switzerland	Review	Scientific publications (*N* = 88)	Literature search	Psychological stress is an independent CHD risk factor associated with increased inflammation
Jacquet-Smailovic et al. (2022)	France	Systematic review and meta-analysis	Patients with PTSD	Literature search	The presence of PTSD is a risk factors for subsequent development of ischemic heart disease, such as MI
Edmondson et al. (2013)	USA	Systematic review and meta-analysis	Patients with PTSD and CHD	Literature search	PTSD is independently associated with increased risk for incident CHD and this association is common in both military veterans and civilian trauma survivors
Kamardeen (2021)	Australia	Structural path analysis	Construction professionals (*N* = 247)	Online questionnaire survey	Junior level professionals reported higher cardiac vulnerability than others. Statistically significant positive associations were discovered between the reported CVDs and job stressors and negative stress coping methods
Watkins et al. (2023)	USA	Randomized clinical trial	Patients with PTSD (*N* = 112)	Cognitive behavioral therapy, interview and 24-h HRV	Effective treatment of PTSD reduces not only psychological distress but also may mitigate significant CVD risk
Gianaros and Jennings (2018)	USA	Review		Literature search	A dysregulation of visceral motor and visceral sensory processes during stressful experiences may confer risk for poor cardiovascular health among vulnerable individuals
Ginty et al. (2017)	USA	Review	Animal models and patient lesion studies	Literature search	Visceral-motor and visceral-sensory mechanisms are involved in cardiovascular reactivity and stress-related CVD risk
Mäenpää et al. (2021)	Sweden	Inductive explorative design	Individuals afflicted by TTS (*N* = 11)	Semi-structured individual interviews 2–12 months after surgery	TTS can lead to changes in life conditions but these changes vary among patients. Becoming ill was associated to acute physical stress, prolonged psychological stress and suffering emotional reactions
Pini et al. (2015)	Italy and Australia	Clinical trial	ACS patients who had experienced the death of a loved one and met the criteria for DSM-5 persistent complex bereavement disorder (*N* = 118)	Questionnaires about anxiety and depression	Among the ACS patients the loss of a close relative and the severity of complicated grief (CG) symptoms are associated with poorer health status, strengthening previous data indicating a strong relationship between CG symptoms and severe cardiac problems
Ennis and Majid (2021)	Canada	Systematic review	Scientific publications (*N* = 38)	Literature search	Most studies have found a widowhood effect, which seems to indicate an increased risk of mortality
Coping strategies and personality traits					
Roohafza et al. (2022)	Iran	Population—based prospective study	6,323 individuals	Self-report questionnaire about adaptive and maladaptive coping strategies, physical exams, and lab data	Coping strategies play an important role in CVD events: adaptive coping strategies are protective, while maladaptive ones could play the same role as a risk factor, particularly in younger adults
Reverté-Villarroya et al. (2020)	Spain	Multicenter, prospective, longitudinal, and comparative study of a sub-study	82 acute ischemic stroke patients (*n* = 42 undergoing EVT and *n* = 40 undergoing BMT as a control group)	Psychological, neurological and functional assessment at 3 months and 1 year after a stroke	EVT patients showed better neurological and functional outcomes, reporting no pain/discomfort at 3 months. On the other hand, problem-focused coping strategies were found to be significantly higher in patients treated with BMT at 1 year
Roohafza et al. (2012)	Iran	Case–control study	78 hospitalized patients with ACS and 146 patients with CSA as the case and control groups, respectively	Interview during hospitalization in 2 separate sessions and questionnaires about life stress events and social support	Adaptive and maladaptive coping strategies associated to the level of social support can play a crucial role in developing or protecting from acute coronary events in chronic IHD patients
Ben-Zur (1999)	Israel	Research	Coronary artery bypass graft surgery (CABG) patients (*N* = 117)	Mail questionnaire two to 20 months after coronary artery bypass graft surgery (CABG)	Postsurgery distress, functional capacity, pessimism, and emotion-focused coping were found to be strongly associated, but it should be remembered that the investigated variables are reciprocally determined
Howard et al. (2017)	Ireland	4 × 1 within-subjects design	86 healthy females	Cognitive stressors and cardiovascular assessment. Post-task rating scales were completed immediately following each task	Repressive coping may be associated with increased risk of cardiovascular disease development through elevated cardiovascular reactions to both novel and recurrent stress
Mustață (2021)	Romania	Transversal, correlational design	274 people with chronic cardiovascular diagnoses	Questionnaires about depression, anxiety and sleep disorders to hospitalized and non hospitalized patients, depending on the severity of the disorder	In cardiovascular patients the levels of depression, anxiety and sleep disorders increase as the levels of self-blame, ruminating and catastrophizing increase and they decrease as the levels of positive refocusing, refocusing of planning and positive reassessment increase
Tung et al. (2008)	Taiwan and United States	Research design	100 post CABG patients	Questionnaires about anxiety, coping and quality of life and qualitative interviews after CABG surgery in the past 5 years	Better quality of life was associated with lower anxiety level, greater use of problem-focused coping strategies and more gender role responsibility
Giuliani et al. (2024)	Italy	Research design	219 women	Psychological questionnaires about depression, anxiety, perceived stress, type D personality assessment and medical data (including arterial blood pressure, conventional and women’s specific cardiovascular risk factors)	Women are still labeled as a special population in many guidelines relating to CVD. Particularly when presenting with acute coronary syndromes, they tend to experience worse outcomes, especially at younger ages
De Fazio et al. (2012)	Italy	Cross-sectional study	81 consecutive post-ACS patients	Patients were assessed as soon as they were medically stable, within 1 week following ACS, through psychological questionnaire about anxiety, depression, coping and Type D personality scale	A high rate of depressive and anxious symptoms was found and 76% of patients resulted Type D personality. Depression was associated with b-blocker therapy, Type D personality, and specific coping strategies. Unmarried status, low education, unstable angina, Type D personality, emotion, and avoidance oriented coping independently predicted anxiety

*ACS (acute coronary syndrome), *BMT (best medical treatment), *CABG (coronary artery bypass graft), *CG (complicated grief), *CHD (Coronary Heart Disease), *CSA (chronic stable angina), *CVD (cardiovascular disease), *CVD (cardiovascular disease), *DSM-5 (Diagnostic and Statistical Manual of mental disorders - 5), *EVT (endovascular treatment), *HRV (heart rate variability), *IHD (Ischemic heart disease), *PTSD (Post Traumatic Stress Disorder), *TTS (Takotsubo syndrome).

Numerous studies have highlighted inflammation as a potential mechanism that underlies the relationship between stress and cardiovascular risk (Wirtz and von Känel, 2017). In this regard, Shimanoe et al. (2014) argue that psychological factors could affect cardiovascular risk through inflammatory reactions that are mediated by C-reactive protein, but the results are not conclusive. Data from prospective epidemiological studies show that chronic stress conditions can lead to a significant risk of CVD and a poor cardiovascular prognosis, supporting the causal relationship between stress and CVD (Steptoe and Kivimaki, 2013). The literature has identified work stress—such as job burnout and vital exhaustion—social isolation, low socioeconomic status, ACEs, caregiving/family stress and PTSD as the most prevalent chronic psychological stressors. Also, acute emotional stress, including trauma feelings and intense negative emotions, can trigger acute CHD events in vulnerable patients (Wirtz and von Känel, 2017).

#### 3.1.1 Workplace stress

Workplace stress has been studied in connection with CVD, and many studies have demonstrated its association with the risk of MI (Welin et al., 1995; Mustață, 2021). Several stressors have been highlighted in the literature with regard to their correlation with work contexts. The cumulative load that is related to chronic stress in this area, known as allostatic load, May in fact be related to various unsafe living situations and working conditions, excessive workload, long working hours, poor job support, interpersonal conflicts, poor job control, workplace bullying and harassment, low job rewards (including low salary), job insecurity, lack of resources, career stagnation, discrimination and work-life conflict (Guidi et al., 2020). In this regard, employees who experience chronic work stress consequently develop increased blood pressure (hypertension), even outside of working hours (Vrijkotte et al., 2000). Moreover, Junior level professionals seem to report higher cardiac vulnerability (Kamardeen, 2021).

According to a biomedical perspective, typical cardiovascular risk factors, including hypertension, high cholesterol, diabetes and obesity, also represent biological markers of allostatic load (The Department of Health, 2022). This finding highlights these markers as mediators between occupational stressors and the development of CVD (Sara et al., 2018). A recent study reported that job stress actually has a negative impact on an individual’s health, increasing blood cholesterol concentrations and body mass index—both of which are risk factors for CVD (Carey, 2020).

#### 3.1.2 PTSD

Many studies (Benjamin et al., 2017) that have been conducted over the past two decades have shown that exposure to certain traumatic events can lead to the development of PTSD, a widespread and disabling mental disorder that occurs following exposure to traumatic events, such as war, intimate partner violence and natural disasters (Boscarino, 2008; Sullivan and Neria, 2009). Common symptoms that are experienced by individuals with PTSD include intrusive thoughts, nightmares, flashbacks, avoidance of memories that are related to the traumatic event and physiological arousal and May lead to an increased risk of suicide, drug and substance abuse and an inability to work (Yehuda, 2006).

Although the profound impact of PTSD on mental health has long been recognized, recent research has increasingly emphasized the negative effects of PTSD on physical health, particularly cardiovascular health (Edmondson et al., 2013). The literature suggests that PTSD can be considered an independent risk factor for CVD—especially ischemic heart disease (IHD) and MI (Jacquet-Smailovic et al., 2022). In this regard, the research highlights a 49% increase in the risk of subsequent onset of MI, MI-related hospitalizations or cardiac mortality after adjustments for several traditional demographic, socioeconomic and cardiovascular risk factors. In addition, PTSD is more likely to correlate with higher risk of long-term hospitalization for MI than short-term hospitalization (Edmondson et al., 2013). The evidence argues that individuals with PTSD tend to engage in behaviors that potentially harm cardiovascular health, such as smoking, a sedentary lifestyle, alcohol consumption and poor diet (Beristianos et al., 2016; Bradley et al., 2014; Crum-Cianflone et al., 2014). Heart rate appears to be a key factor in the relationship between PTSD and the risk of adverse cardiac events (Watkins et al., 2023; Jacquet-Smailovic et al., 2022). Hyperarousal, a characteristic of PTSD, is involved through the excessive and chronic activation of the stress response (Kubzansky, 2009). Individuals with PTSD experience protracted increases in heart rate and prolonged withdrawal of parasympathetic nervous system activity (Watkins et al., 2023). The literature also indicates that the prevalence rates of known risk factors for cardiovascular disorders, such as obesity, diabetes, dyslipidemia and hypertension, are significantly higher in this clinical population (Jacquet-Smailovic et al., 2022).

#### 3.1.3 TTS

A specific link exists between emotional stress and the onset of Takotsubo syndrome (TTS), first described in Japan in 1990 and also known as “broken heart syndrome,” “stress cardiomyopathy” and “apical balloon syndrome” (Sharkey et al., 2011). TTS predominantly affects women, who are at higher risk compared with men; women with TTS tend to have an average age of 67–70 years (Deshmukh et al., 2012). A study has suggested that emotional stress, such as sadness, anger, fear and conflict, is a significant trigger for TTS in women, whereas physical stress is a more significant factor in men (Sharkey et al., 2011).

Recent research (Deshmukh et al., 2012; Wallström et al., 2016) indicates that TTS patients often underestimate the emotional impact of a triggering event, referring to people with such personality traits as perfectionism and helpfulness—qualities that appear to be involved in the response to a stressful event.

Considering these findings, chronic stress should be considered a potential risk factor for TTS (Mäenpää et al., 2021), in addition to the coping strategies that individuals use to deal with stress.

#### 3.1.4 Bereavement

Most people, at some point in their lives, lose a parent, spouse, sibling or child, and the loss of a loved one can provoke endure stressful events in them. Experiencing the loss of a spouse, known as spousal bereavement, is linked to negative health consequences, including a heightened risk of acute cardiovascular events and higher mortality rates, especially within the first 6 months following the loss of a spouse (Carey et al., 2014).

The loss of a spouse is among the many traumatic events that can disrupt an individual’s psychological balance, causing psychological and existential distress (Majid and Ennis, 2018; Ennis and Majid, 2021). People who grieve May develop a form of excessive and prolonged grief—referred to as complicated grief (CG)—that interferes with daily activities (Mason and Duffy, 2018). This condition is also known as persistent complex bereavement disorder and is mentioned in the Diagnostic and Statistical Manual of Mental Disorders (Lichtenthal et al., 2004; Prigerson et al., 2009; Shear et al., 2011; American Psychiatric Association, 2013). CG is characterized by such symptoms as longing for the deceased, intense grief, preoccupation with the circumstances of death, recurring intrusive thoughts of death, disbelief, avoidance of memories of the deceased and a sense of life’s meaninglessness. These symptoms can persist for over 1 year after the loss (Horowitz et al., 1997; Boelen and van den Bout, 2008; Shear and Shair, 2005).

Some studies suggest that CG has a negative impact on physical health and chronic conditions (Prigerson et al., 1997; Kristensen et al., 2015; Dell’Osso et al., 2011). In particular, Prigerson et al. (2009) have proposed an association between CG symptoms and the risk of developing heart problems. In support of this relationship, Pini et al. (2015) noted that a large percentage (79.2%) of individuals who experienced an acute MI (ACS—acute coronary syndrome), with no prior history of previous CHD, had suffered from the loss of a loved one and showed symptoms of persistent and CG (Pini et al., 2015). They also reported that the loss of a partner, child or sibling was associated with the presence of hypertension and worse health status compared with that of a parent or other relative. Thus, the nature and quality of the relationship between a subject and his deceased relatives are important factors of cardiovascular health that merit further examination.

For example, the loss of a child is one of the most distressing experiences in life (Li et al., 2005; Wijingaards-de Meij et al., 2007). Parents who have lost a child seem to endure more severe CG than individuals who have experienced another type of loss. Further, the severity of CG symptoms correlates positively with the onset of ACS 12–48 months after the loss and inversely with that after this period (Sveen et al., 2014; Ghesquiere et al., 2012)—a temporal relationship between CG and the occurrence of a first acute coronary episode that the authors highlighted as a notable finding (Pini et al., 2015). Consistent with this relationship, a span of at least 12 months (6 months in children) after the loss discriminates normal pain from persistent pain.

Individuals who experience prolonged bereavement report higher dysregulation of cortisol levels (Ennis and Majid, 2021; Mason and Duffy, 2018). However, there are little data on blood pressure, inflammatory changes, and physiological mechanisms that accompany this phenomenon.

### 3.2 Coping strategies and personality traits in cardiovascular disease

#### 3.2.1 Coping strategies

Coping strategies consist of behavioral and cognitive efforts by an individual to deal with stressful events (Lazarus and Folkman, 1984) and vary among people, based on their ability to control stress (Roohafza et al., 2022). Coping strategies, which are associated with a better quality of life, have two major functions: addressing the problem that is causing the distress (problem-focused coping) and regulating emotion (emotion-focused coping) (Yu et al., 2013). Specifically, problem-focused strategies are considered to be more adaptive, because they entail the implementation of active behaviors and strategies that are aimed at changing or resolving the problem (Lazarus and Folkman, 1984; Carver et al., 1989), such as learning new skills, seeking help and venting anger (Kristofferzon et al., 2003; Kristofferzon et al., 2005).

Conversely, motion-focused strategies are designed to alter or manage the affective and physiological outcomes of the stressful situation, without actively acting on the event itself (Lazarus and Folkman, 1984), and are assumed to refer to situations that are considered unmanageable (Ben-Zur, 1999; Carver et al., 1989). The latter include rumination, aggression and passive avoidance (Kristofferzon et al., 2003; Kristofferzon et al., 2005) and, for instance, appear to be maladaptive.

In the literature, coping strategies have been evaluated as relevant psychological factors in patients with coronary involvement (Table 1). According to various studies (Roohafza et al., 2012; Roohafza et al., 2022), problem-focused coping strategies protect against CVD events and are associated with a lower incidence of stroke and CVD-related mortality, whereas emotion-focused approaches might constitute a risk factor, especially in younger adults. Emotion-focused strategies also correlate with a worse recovery after stroke (Tramonti et al., 2014; Lyon, 2002). In particular, negative emotions, such as anger, guilt, sadness and avoidant behavior that is related to an unexpected event (for example, a stroke), evoke helplessness and consequently less acceptance of it. All of these effects can contribute to recurrent additional stress that prevents the development of healthy behaviors (Lyon, 2002; Donnellan et al., 2006; Sirois et al., 2015).

Moreover, frequent use of emotion-focused coping strategies has been associated with an increased risk of hypertension (Ariff et al., 2011), whereas the use of problem-focused coping is linked to lower blood pressure and lower pulse (Mustață, 2021). In a study that investigated the coping strategies between patients in the acute phase of stroke recovery, based on the treatment that was received—endovascular therapy (EVT) or standard best medical treatment (BMT)—those who were administered BMT had significantly higher scores in active coping, as evidenced by their more positive attitudes toward their condition, manifesting as accepting the illness and finding solutions to improve their status (Lazarus et al., 1986). Conversely, although EVT-treated patients had better functional and neurological outcomes, less clinical invasiveness was related to their lower perception and awareness of the illness, implying less coping and acceptance by the patients (Reverté-Villarroya et al., 2020).

Mechanisms that link coping strategies and CVD in patients with ACS could be postulated to underpin the association between emotion-focused coping strategies and related risk factors, such as higher blood pressure (Theorell, 1996), increased waist-to-hip ratio and altered lifestyle—for example, reduced daily physical activity, poor social support and greater alcohol consumption (Twisk et al., 2000). Regular exercise is an adaptive coping strategy that promotes an anti-inflammatory state, stimulates myocardial regeneration and improves age-related loss of muscle mass and strength, a non-typical CVD risk factor that is often ignored (Fiuza-Luces et al., 2018). Individuals with a repressive coping strategy tend to report less negative emotion and to describe challenges as being less stressful but have higher rates of CVD (Howard et al., 2017). According to a recent study, repressors had elevated cardiovascular responses to new and recurrent stresses compared with non-repressors, suggesting that repressive coping is associated with exaggerated cardiovascular responses to stress (Schwerdtfeger et al., 2005), with maladaptive responses persisting on repeat exposure, despite some degree of cardiovascular habituation (Howard et al., 2017).

An avoidance-coping strategy in patients with pre-existing CVD risk factors, such as hypertension, appears to correspond to maladaptive coping strategies and higher mortality among patients with CVD (Roohafza et al., 2022). Further, this type of repressive coping, termed cognitive avoidant coping (CAV) by the authors, has been linked to elevated autonomic stress reactivity and could constitute a risk factor for CVD with increasing age. Specifically, CAV seems to address elevated blood pressure, heart rate and skin conductance. The relationship between avoidant coping strategies and intima media thickness (IMT), considered a surrogate marker of atherosclerosis that elevates the future risk for CVD, has been examined in the literature. In older individuals, elevated CAV scores are accompanied by greater IMT (Schwerdtfeger et al., 2015).

The link between coping, anxiety, depression, quality of life and CVD has also been studied in detail. According to the literature, emotion-focused coping strategies that correlate with these negative emotional pathologies include self-blaming, ruminating and catastrophizing, whereas problem-focused approaches consist of positive refocusing, positive reassessment and refocusing on planning (Mustață, 2021). Anxiety and depression have been associated with avoidant coping strategies (Finset and Andersson, 2000; Sinyor et al., 1986), less behavioral action and fewer rational cognition strategies (Gillespie, 1997). Specifically, depression is also related to the use of magical thinking coping domains (Gianaros et al., 2009). A study of post-coronary artery bypass graft (CABG) patients suggests that problem-focused thinking behavior decreases their sense of anxiety and correlates with a better quality of life. In patients, the act of focusing on addressing the problem itself is considered a positive emotional reaction (Tung et al., 2008). Moreover, anxiety and amygdala reactivity are associated with a higher IMT (Schwerdtfeger et al., 2015), thus favoring that an anxious and hypervigilant disposition is a reliable risk factor for CVD (Schwerdtfeger et al., 2015).

#### 3.2.2 Personality traits and cardiovascular outcomes

Individuals with Type-D personality exhibit a pattern of psychological distress, characterized by persistent negative emotions and a reluctance to engage socially (Denollet et al., 2006; Pedersen and Denollet, 2006). This personality trait has a significant impact on various outcomes in cardiac patients (Table 1) (Giuliani et al., 2024). Patients with Type-D personality implement emotion-focused coping strategies in response to disease, such as resignation and withdrawal, which predicts morbidity and mortality in coronary vascular disease and ultimately impairs the quality of life and their subjective well-being (Tung et al., 2008). Consequently, an adverse prognosis in patients with coronary artery disease (CAD), including a doubled risk of mortality and nonfatal myocardial infarction (MI) (Tung et al., 2008), poor adherence to treatment (Giuliani et al., 2024), difficulties in performing health-promoting behaviors (Tung et al., 2008) and decreased self-esteem can worsen the condition. Additionally, type-D personality is a predictor of worse health status in CAD and heart failure (HF) patients, and is linked to lower quality of life in CAD patients undergoing rehabilitation. Furthermore, it is associated with poor treatment adherence and higher serum inflammatory markers (Staniute et al., 2015). Type-D personality traits, specifically negative affectivity and social inhibition, are strongly correlated with both depression and anxiety (Mols and Denollet, 2010). A study conducted by De Fazio et al. (2012) on affective symptoms in early post-ACS patients showed a high percentage of depressive and anxious symptoms in the sample, correlated with a type D personality and a high level of emotionally oriented coping (Lazarus and Folkman, 1984). It is plausible that these traits May increase susceptibility to the development or worsening of anxious and depressive symptoms, thereby contributing to an increased risk of cardiovascular events. A recent study found that patients with Type-D personality who underwent percutaneous coronary intervention showed a significantly higher risk of depression and anxiety at 10-year follow-up (Al-Qezweny et al., 2016).

### 3.3 Early life psycho-social stress and CVD

#### 3.3.1 Adverse childhood experiences

Individuals May encounter various ACEs, especially in the context of caregiving. These negative experiences include a wide range of forms of violence and maltreatment. Due to the frequent concurrence of multiple types of child maltreatment, the literature often labels this phenomenon “multitype child maltreatment,” as described by Higgings and McCabe (2001).

ACEs refer to stressful or traumatic experiences—such as sexual, emotional and physical abuse; physical and emotional neglect; and alcoholism or psychiatric illness in the family—that are characterized by threat, unpredictability or deprivation (McLaughlin et al., 2021). ACEs have many consequences on development (Brindle et al., 2022). In general, having experienced four or more ACEs is associated with an increased risk of adverse health outcomes in adulthood (Hughes et al., 2017), and ACEs have been linked to a reduced quality of life and subsequent mental disorders (Rinnewitz et al., 2018). Among their serious biological effects, the literature highlights elevated inflammation (Matthews et al., 2014; Nemeroff, 2016), structural and functional changes in the developing brain—for example, in amygdala and hippocampal reactivity (Ginty, 2019)—and gene expression and epigenetic changes (Şimşek et al., 2016).

Moreover, having been abused in childhood May increase the risk of engaging in dysfunctional behaviors, such as alcoholism, smoking and substance abuse (Suglia et al., 2018; Gillespie, 1997), High-risk health behaviors, some of which independently contribute to the development of cardiovascular disease (Merrick et al., 2018).

ACEs, including domestic abuse, physical abuse and sexual abuse, have been identified as significant risk factors for the development of acquired CVD (Jacquet-Smailovic et al., 2022; Table 2) and increased mortality (Felitti et al., 1998). The interplay of biological and behavioral factors can directly or indirectly increase the risk of CAD, often exacerbating established risk factors, such as hypertension (Stein et al., 2010), obesity (Matthews et al., 2014), elevated cholesterol levels (Kreatsoulas et al., 2019) and diabetes (Andrade et al., 2021)—all of which are independent risk factors for CVD. In addition, the connection between ACEs and CVD is well established (Chen et al., 2023; Bellis et al., 2015), as evidenced particularly by a heightened risk of MI (heart attack) in adulthood (Jacquet-Smailovic et al., 2022; Bellis et al., 2015; Hughes et al., 2017).

**Table 2:** Selected studies describing the implication of adverse childhood experiences and attachment in CVDs.

Authors (year)	Country	Study design	Population	Methods	Outcomes
Adverse childhood experiences and attachment					
Andrade et al. (2021)	Florida	Cross-sectional study	133 Hispanic adolescents	Medical records and survey	Participants who experiences ≥4 ACEs show risk markers of obesity and cardiometabolic disease
Basu et al. (2017)	/	Review	40 scientific publications	Literature search	The association between child maltreatment and cardiometabolic disorders was moduled by gender
Brindle et al. (2022)	/	Meta-analysis	83 scientific publications	Literature search	ACEs is linked to diminished cardiovascular and cortisol reactivity. ACEs may trigger physiological adaptations that could be advantageous in the short term but harmful to long-term health
Brown et al. (2021)	/	Review	37 scientific publications	Literature search	There is not a predominantly significant connection between childhood trauma and inflammatory markers in adulthood. However, there are noteworthy differences based on the type of trauma, the specific inflammatory marker considered, and other relevant variables
Caceres et al. (2022)	United States	Cross-sectional substudy	1,251 adults	Questionnaire	Depressive symptoms, sleep quality and sex were explored as potential mediators in the relationship between childhood trauma and cardiovascular health
Chen et al. (2023)	/	Systematic review and meta-analysis	10 scientific publications	Literature search	Child abuse is associated with an increased risk of CHD in the adult. Results are generally consistent by type of abuse and by gender
Flores-Torres et al. (2020)	Mexico	Prospective study	9,853 Mexican Teacher’s Cohort	Questionnaire	ACEs may independently be associated with dysfunctional behaviors and health conditions linked to cardiovascular health in adulthood
Heenan et al. (2020)	Canada	Clinical Trial	201 patients in a cardiac rehabilitation program	Physiological and psychosocial variables at program intake and end of program, attachment and traumatic stress at baseline only	Childhood maltreatment experiences could lead to cardiovascular disease risk
Jakubowski et al. (2018)	/	Review	37 scientific publications	Literature search	Cumulative childhood adversity is modestly related to adult cardiometabolic disease. A consistent operational conceptual definition of adversity is needed
Kidd et al. (2014)	United Kingdom	Clinical Trial	167 CABG surgery patients	Questionnaire at surgery. Blood samples at and (1–3 days) after surgery	Increased IL-6 concentrations after surgery were associated with anxious attachment and low sleep quality
Kidd et al. (2016)	United Kingdom	Clinical Trial	155 CABG surgery patients	Questionnaire at surgery and (6–8 weeks; 12 months) after surgery	High levels of anxious and avoidant attachment were associated with high levels of depression and anxiety symptoms at follow‐ups. Social support was negatively associated with both dimensions of attachment
Lee et al. (2023)	/	Cross-sectional design	31,242 individuals	Network analysis	The variable “domestic incarceration” showed a solid association with stroke in men. In women there is a strong association between “physical abuse” and stroke and “sexual abuse” and CHD
McWilliams and Bailey (2010)	Canada	Cross-sectional study	5,645 participants from the National Comorbidity Survey Replication	Diagnostic assessment of psychiatric disorders and chronic health conditions. Interview on adult attachment style	Avoidant attachment positively associated with conditions characterized by pain. Anxious attachment positively associated with health conditions involving the cardiovascular system
Meredith and Strong (2019)	/	Review	/	Literature search	Factors associated with insecure attachment represent risk factors for the development of chronic health conditions. A dismissing pattern has been associated with mortality
Peleg et al. (2017)	Israel	Prospective longitudinal design	106 ACS male patients	Questionnaire including attachment orientation during hospitalization and questionnaire measuring adherence to medication (6 months) post-discharge	Link between perceived behavioral control and adherence in cases of low attachment anxiety, attitudes and intention in cases of high attachment anxiety, subjective norms and intention to take medication among individuals with high attachment avoidance
Pietromonaco and Beck (2019)	/	Meta-Analysis	45 scientific publications	Literature search	Attachment insecurity in adulthood linked to poor physical health. Insecure attachment associated with distinctive cardiovascular responses to stress
Pietromonaco and Collins (2017)	/	Meta-Analysis	101 scientific publications	Literature search	Close relationships affect health conditions. Attachment insecurity may moderate the link between social connection/disconnection and biological responses to stress, health behaviors, and health outcomes
Pietromonaco et al. (2013)	/	Meta-Analysis	114 scientific publications	Literature search	Attachment theory offers insights into both normative processes of careseeking and caregiving that are significant in the context of health threats and individual differences in attachment style that can shape individuals’ health behaviors and outcomes
Robles and Kane (2014)		Review	/	Literature search	Insecure attachment may have implications for restorative processes and it is associated with sleep disturbance. Disclosure to intimate partners may attenuate the disturbances
Suglia et al. (2018)	United States	Review	scientific publications	Literature search	59% of the US population reports at least one ACEs
Vilchinsky et al. (2010)	Israel	Prospective study	111 ACS patients and their partners	Questionnaire about attachment orientations during hospitalization by patients, about ways of providing support 1 month later by spouses. Questionnaire about psychological distress of patients 6 months after ACS	Anxious-depressive symptomatology in patients 6 months after the cardiac event. Active involvement associated with low anxiety if high anxious attachment, and with high anxiety if low anxious attachment. Providing support not moderated link between avoidance and distress

*CHD (coronary Heart disease); *ACEs (Adverse childhood experiences); *CABG (coronary artery bypass graft); *ACS (acute coronary syndrome); *IL-6 (Interleukin - 6).

The literature highlights the significance of specific childhood experiences and emphasizes the need for further research (Brown et al., 2021). Specifically, physical abuse and sexual abuse are identified as major risk factors for the development of CVD (Flores-Torres et al., 2020). In addition, there is a consistent link between physical abuse in children and a greater risk of CVD, especially stroke (Basu et al., 2017).

Gender appears to influence the impact of ACEs on CVD. Some studies have reported greater vulnerability and stronger associations between ACEs and CVD in women (Hosang et al., 2013; Lee et al., 2023). Consistent with these findings, Lee et al. (2023) note that among individuals who have experienced physical and sexual abuse, women and older people are more susceptible to CVD. However, others have generated contradictory results, in which women have had more favorable CVD profiles (Felitti et al., 1998). Flores-Torres et al. (2020) identified robust associations between ACEs and independent risk factors for cardiovascular disorders, particularly in women who reported experiencing four or more ACEs.

Neglect has been closely linked to hypertension, whereas physical abuse and sexual abuse have emerged as primary risk factors for CVD; these forms of abuse are also strongly associated with regular alcohol consumption. This result thus considers the complex association of known risk factors for CVD (Andrade et al., 2021).

Relevant traumatic events that have been considered risk factors for the development of CVD include sexual violence (Jakubowski et al., 2018) and the impact of war (Haas and Ramirez, 2022). Exposure to war is a frequent occurrence in many regions worldwide. Several studies (Haas and Ramirez, 2022; Kadir et al., 2019) have shown that children who experience armed conflict suffer negative short-term health consequences, including cardiometabolic disorders.

ACEs can alter stress regulatory processes, and prolonged exposure to stressful life events can trigger changes in metabolic, immune and neuroendocrine responses. Such disruptions May involve factors, including diabetes and hypertension, which represent intermediate steps in the complex pathway that bridges stress and CVD (Steptoe and Kivimaki, 2013). Childhood experiences shape the attachment style in the adult, understood as a set of strategies for responding to stress and fear (Mikulincer and Shaver, 2007). There is a dearth of studies that have examined the impact of attachment style in cardiology, although attachment style has been shown to impact physical health, particularly cardiovascular risk (McWilliams and Bailey, 2010; Heenan et al., 2020).

#### 3.3.2 Attachment

The available evidence (Pietromonaco et al., 2013; Robles and Kane, 2014; Meredith and Strong, 2019; McWilliams and Bailey, 2010; Heenan et al., 2020; Kidd et al., 2016; Pietromonaco and Collins, 2017; Pietromonaco and Beck, 2019) converges in determining that attachment style affects health status. In this regard, childhood attachment May influence the style that develops in adulthood but also the specific responses to stress (Pietromonaco and Collins, 2017; Pietromonaco and Beck, 2019).

Individuals with a secure attachment model seek support from a caregiver and expect him or her to be available to respond to their needs. In contrast, those who present with avoidant attachment assume that their partners are less responsive and thus tend to manage the threat by minimizing their suffering and withdrawing from others. Individuals who express anxious attachment fear that their partners are unavailable, leading them to react by insistently pointing out their distress and looking for excessive support.

The quality of early interpersonal experiences in childhood shapes the self-regulatory skills of individuals throughout their lives (Bowlby, 1958). Insecurely attached persons cannot perceive the security and comfort of attachment figures and are more likely to feel uncomfortable in these relationships (Mikulincer and Shaver, 2007). Further, insecure attachment has been shown to be a risk factor for the development of chronic health conditions (Pietromonaco et al., 2013; Robles and Kane, 2014; Meredith and Strong, 2019), particularly those that involve the cardiovascular system (McWilliams and Bailey, 2010; Heenan et al., 2020). No useful data on the disorganized attachment style appear to be available among published studies.

Meaningful interpersonal relationships can be a resource for protecting and promoting health, owing to mechanisms of social integration and perceived support, but they can also be a potential cause of stress, through interactions that are characterized by hostility and social rejection (Pietromonaco and Beck, 2019). In addition to hypothalamic–pituitary–adrenal (HPA) axis activity and immune responses, specific cardiovascular system responses have been identified among biological reactions to stress that are related to attachment style in adults (Pietromonaco and Beck, 2019). The data are conflicting for individuals with more avoidant partners, who experience a greater physiological threat yet report lower cardiovascular reactivity and thus a lower risk of disease onset. Individuals with an anxious partner generally have higher cardiovascular reactivity, which decreases when they receive support during interactions (Pietromonaco and Beck, 2019). Unlike avoidant attachment, the insecure-anxious style correlates with several disorders, including stroke, heart attack and high blood pressure (McWilliams and Bailey, 2010).

Consistent with these outcomes, other studies support the hypothesis that attachment anxiety affects physical recovery after surgery (Kidd et al., 2016) and that it predicts anxiety-depressive symptoms in patients who have experienced cardiac surgery, in the short and long term (Kidd et al., 2016).

In contrast to individuals with a secure attachment style, those with insecure attachment tend to be more vulnerable to the effects of potentially traumatic experiences, such as cardiac events (Heenan et al., 2020). In particular, the avoidant style is non-predictive of traumatic stress; this result can be explained by the tendency to underestimate the impact of the event, rather than by the individual’s resilience to trauma. Instead, anxiously attached individuals have less effective tools for perceiving and integrating the emotional support that they receive from caregivers. These findings indicate that greater attachment anxiety predicts higher traumatic stress, which correlates with worse health outcomes (Heenan et al., 2020). Attachment anxiety is associated with an increase in anxiety symptoms, and for these patients, active engagement by the caregiver improves the management of their symptoms (Vilchinsky et al., 2010).

Individuals with secure attachment typically value and use social support more than those with anxious attachment, perhaps because the perception of an external network provides the psychological security to invoke self-reliant coping strategies. Patients with an avoidant style search for less support from their partner when they are faced with severe stress, because they have learned to cope with it through self-reliance. It has been hypothesized, however, that patients with an insecure-anxious style benefit from the support that they receive from a caregiver, because their need for dependence is satisfied (Vilchinsky et al., 2010).

In patients who have been treated for cardiac problems, no significant direct association has been found between attachment style and intentions or behaviors with regard to adherence to medical treatment (Peleg et al., 2017). Attachment, however, May moderate these mental processes, particularly the insecure-anxious orientation, wherein strongly anxiously attached individuals have worse compliance (Peleg et al., 2017).

### 3.4 Anxious-depressive symptoms in CVD

In light of their established comorbidity both in the general population and in CVD patients, in this review we report findings considering both conditions as a continuum rather than as two distinct nosographic entities.

Prior to surgical interventions, patients who experience anxiety symptoms May be at risk for greater postoperative pain and more pronounced physical symptoms, even up to 12 months following surgery (Martens et al., 2010). In stable CHD, individuals with generalized anxiety disorder face a 74% higher rate of cardiovascular events, and this association is not affected significantly by the presence of major depressive disorder (Aw et al., 2023; Table 3). After cardiac surgery or a cardiac event, the existence of anxiety symptoms has been linked to a heightened risk of cardiac-related readmission and poorer outcomes post-infarction (Oxlad et al., 2006; AbuRuz et al., 2021). Depressive symptoms before surgery are more frequent in female patients (Wu and Kling, 2016; Karami et al., 2023) and have been associated with various factors, including a higher likelihood of emergency admissions, an increased hazard of death or major adverse cardiovascular events and a greater risk of cardiac-related readmissions (Martens et al., 2010), with a significant increase in the risk of MI and coronary death (Horne et al., 2013). These symptoms can also portend the development of post-surgical depression and extended hospital stays (Martens et al., 2010; Liu et al., 2019; AbuRuz, 2019; Oxlad et al., 2006).

**Table 3:** Selected studies describing the implication of anxious and depressive symptoms in CVDs.

Authors	Country	Study design	Population	Methods	Outcomes
AbuRuz (2019)	Jordan	Prospective observational study	227 CABG patients	Questionnaire (2 weeks) before and (1 month) after surgery	Pre-operative depressive symptoms showed in 42.74% of the patients. High symptoms prolonged LOS
AbuRuz et al. (2021)	Jordan	Prospective cohort study	220 CABG patients	Questionnaire (2 days) before surgery	Females exhibited higher levels of depressive symptoms than males and prolonged LOS. High prevalence of preoperative depression among sample and its association with increased postoperative hospitalization. Moderating role of perceived control
Ai et al. (2012)	Unites States	Non-experimental research design	235 participants	Interview (2 weeks) before surgery. Blood samples after surgery (3 days)	LOS is correlated with old age, medical comorbidities, perfusion time, and postoperative IL-6
Aw et al. (2023)		Review	57,342 patients	Literature search	In patients with depression, the pooled prevalence of stroke was 5.9%. In patients with MI, the pooled prevalence of anxiety and depression was 9.1 and 25.9%, respectively, and the pooled cumulative incidence of depression at 1 year was 20.5%. The pooled prevalence of anxiety and depression in patients with stroke was 13.5 and 23.0%, respectively. The pooled cumulative incidences of depression at 2 weeks, 3 months, 6 months, and one year, were 29.1, 17.0, 7.4, and 9.1%, respectively
Bradley and Rumsfeld (2015)		Review	CVD patients	Best practices for managing and treating CVD patients with depression	Patients with depression and CVD can improve through pharmacologic, psychotherapy, and collaborative care interventions. Treatment results in better physiologic markers, lower depression severity, and higher quality of life
Caspi-Avissar et al. (2021)	Israel	Prospective study	100 patients	Questionnaire before surgery, during hospitalization and 2 and 6 weeks after discharge	Depression score levels increased from preoperative to hospitalization and decreased at 2 weeks and 6 weeks after discharge
Dougherty et al. (2016)	United States	Randomized clinical trial, longitudinal study	168 patients	Questionnaires for patient–partner dyads at hospital discharge and at 1, 3, 6 and 12 months follow up	Patient general health improving and physical symptoms decreasing across the recovery period. In contrast, partner general health declining slightly during the same period. Partners compared to patients, reported higher levels of anxiety and depression during the first year
Gorini et al. (2022)	Italy	Explorative study	151 participants	Questionnaire regarding anxiety-depressive symptomatology before and measuring quality of life (3 months) after surgery	Preoperative anxiety predict the LOS, and both anxiety and depression symptoms predict a quality of life after surgery
Horne et al. (2013)	Canada	Longitudinal study	436 patients	Questionnaires for depression and physical activity preoperatively, at hospital discharge, and postoperatively (3 months, 6 months)	Up to 40% of patients are depressed postoperatively. Preoperative depression and postoperative stressful events predict postoperative depression. Physical inactivity was associated with preoperative depression and new depression 6 months postoperatively
Karami et al. (2023)		Review	182,593 patients	Literature search	The overall estimation of the prevalence of depression was 31.3%, anxiety prevalence; 32.9% and stress prevalence was 57.7%
Karlsen et al. (2021)		Review	CVD patients	Literature search	The current standing of anxiety as an independent risk marker of CVD is ‘possible’, and should not be treated interchangeable with depression, despite their co-morbidity
Kidd et al. (2014)	United Kingdom	Clinical Trial	167 CABG surgery patients	Questionnaire at surgery. Blood samples at and (1–3 days) after surgery	Increased IL-6 concentrations after surgery were associated with anxious attachment and low sleep quality
Lichtman et al. (2008)	/	Review	CHD patients	Multispecialty consensus document	Screening, taking care of symptomatic patients, monitoring adherence to treatments and coordination between the various healthcare providers are effective
Liu et al. (2019)	United States	Longitudinal study	34,653 patients	Analysis of longitudinal data from the National Epidemiologic Survey on Alcohol and Related Conditions (NESARC) conducted in 2001/2002 (Wave 1) and 2004/2005 (Wave 2) with additional interviews	Existence of MDD/GAD, MDD, or GAD increase the risk of new-onset CAD. Positive change in MDD and GAD is associated with reduced risk of incident CAD
Martens et al. (2010)	Netherlands	Prospective cohort study	1,015 patients	All the participants completed a baseline examination that included an interview, fasting venous blood sample collection, a psychiatric interview, a questionnaire, echocardiography, exercise treadmill testing, 24-h ambulatory electrocardiography, and 24-h urine collection. Monitored for CVD-related hospitalizations for 9 years after	After adjusting for demographic characteristics, comorbid conditions (including major depressive disorder), cardiac disease severity, and medication use, GAD remained associated with a 62% higher rate of cardiovascular events
Mihandoust et al. (2021)	United States	Quantitative exploratory study	652 participants	Nationwide online survey among patients who received care in hospitals within the previous year	The presence of windows, access to views from the bedside, and views of green spaces all had a significant impact on hospital satisfaction ratings and perceived quality of care
Oxlad et al. (2006)	Australia	Prospective observational study	119 CABG patients	Interviews 1 week after cardiac surgery diagnosis, an average of 50 days prior to surgery	Pre-operative psychological factors influenced post-operative ones. High depression and low PTSD symptomatology identified as significant independent risk factors for prolonged LOS
Poole et al. (2017)	United Kingdom	Explorative study	251 patients	Presurgical assessment of depression and anxiety. Follow-up at 12 months for pain, physical symptoms, and emergency admissions, death and MACE were monitored till 2.68 years after	Anxiety symptoms were associated with greater pain and greater physical symptoms 12 months after surgery whereas depression symptoms were associated with emergency admissions and greater hazard of death/MACE
Rumsfeld et al. (2003)	United States	Multicenter prospective cohort study	460 patients	Questionnaires on depression and cardiomiopathy both at baseline and follow-up (6 +/−2 weeks)	Depressed patients were at risk for significant worsening of their HF symptoms, physical and social function, and quality of life. Depressive symptoms were the strongest predictor of decline in health status in the multivariable models
Tully et al. (2014)		Review	Patients with and without CHD	Literature search	Worry and GAD are associated with blood pressure and diagnosed hypertension or medication use in both disease-free and established CHD populations. No evidence supports worry as a protective factor. GAD is associated with poorer prognosis in established CHD, independent of depression and is marginally less common in CHD samples than depression
Tully et al. (2016)		Review	CHD patients	Literature search	Anxiety portends adverse prognosis in persons with established CVD that is independent from depression
Ulrich (1984)	United States	Qualitative exploratory study	46 participants	Information recorded by nursing staff during hospitalization	Patients with the tree view had brief LOS and low postsurgical complications, they had few negative evaluative comments from nurses and took less analgesic doses
Vaccarino et al. (2001)	United States	Prospective study	391 patients	Questionnaires on depression and activities in daily living, at baseline and at follow-up (6 months)	Baseline depressive symptoms in patients with HF are associated with functional decline and death at 6 months
Wu and Kling (2016)		Review	19 scientific publications	Literature search	Prevention and treatment of depression may substantially decrease the risk of MI and coronary death globally

*CABG (coronary artery bypass graft); *ACS (acute coronary syndrome); *LOS (Length of hospital stay); *PTSD (Post Traumatic Stress Disorder); *MDD (major depressive disorder); *HF (Heart Failure); *GAD (General Anxiety Disorder); *MI (Myocardial infarction); *CVD (cardiovascular disease); *CHD (coronary heart disease); *MACE (Major adverse cardiac events); *CAD (coronary artery disease).

Following an acute cardiovascular event or surgery, up to 40% of patients May experience depressive symptoms (Liu et al., 2019). These symptoms can be predicted by postoperative stressful events and are strongly associated with negative outcomes, such as postoperative delirium, unplanned hospital admissions, cardiac events, infections, impaired wound healing, reduced emotional and physical recovery and diminished quality of life (Martens et al., 2010). Both depressive and anxious symptoms elevate the risk of new-onset CAD 5 years after their initial assessment. Further, depression scores tend to rise during hospitalization but decrease within the weeks following discharge (Horne et al., 2013; Liu et al., 2019; Tully et al., 2014; Caspi-Avissar et al., 2021; Dougherty et al., 2016). Many studies have shown that depressive symptomatology is associated with and predicts worse outcomes following hospitalization for CVD. CVDs encompass myriad manifestations, and the outcomes of various cardiovascular events May be inauspicious if they are associated with depression, including acute MI (Bradley and Rumsfeld, 2015), ACS, ischaemic heart disease, stable ischaemic heart disease (post-CABG) and heart failure (Bradley and Rumsfeld, 2015). In addition, a relationship between severity of depression and cardiovascular outcomes has been observed. Although not all published studies suggest that depression is associated with worse outcomes after ACS, the preponderance of evidence led to a scientific statement by the American Heart Association, defining depression as a formal risk factor for adverse outcomes in patients with ACS (Lichtman et al., 2008).

In addition to being associated with worse survival and increased risk of recurrent cardiovascular events, depression is strongly linked to worse patient health status (i.e., symptom burden, functional status and quality of life) and thus worse perceived health status over time (Bradley and Rumsfeld, 2015). Further, beyond its correlation with worse cardiovascular-specific measures of health status, depression predicts declining health status. In a study of 460 outpatients with a history of heart failure, depression was the strongest predictor of a decline in health status over a 6-week follow-up period (Rumsfeld et al., 2003). These results are consistent with a study by Vaccarino et al. (2001), who identified a stepwise relationship between the severity of depressive symptoms and functional decline or death in heart failure patients.

Depressive symptomatology, in particular, has emerged as a prevalent psychological factor that affects recovery from acute cardiac events (Oxlad et al., 2006; Kidd et al., 2014; AbuRuz, 2019; AbuRuz et al., 2021). This relationship operates in a circular manner, with pre-operative depressive symptoms contributing to a longer length of stay (LOS) and post-operative depression negatively impacting the patient’s ability to recover (AbuRuz, 2019). Some researchers argue that whereas anxiety-depressive symptomatology negatively affects a patient’s quality of life in the months following cardiac surgery, it is primarily anxiety, not depression, that predicts increases in post-operative LOS (Gorini et al., 2022). This finding suggests that the specific manifestation of psychological distress determines its impact on LOS.

Attachment anxiety has been implicated as an independent predictor of hospital stay in cardiovascular patients, whereas attachment avoidance does not appear to have the same effect (Kidd et al., 2014). Patients who report higher levels of attachment anxiety are more susceptible to anxiety-depressive symptoms, and this predisposition May compromise their short-term and long-term recovery following cardiac surgery (Kidd et al., 2014). In addition, the structure of the hospital room has long been known to influence the patient’s experience in post-operative recovery, wherein the room design, bed layout and especially the presence of windows with views of vegetation promote a better perception of care and a shorter LOS (Mihandoust et al., 2021; Ulrich, 1984).

Despite the broad consensus of the potential risk to which a cardiovascular patient with depression as a comorbidity is exposed, the few studies that have controlled for the effect of depressive symptomatology to examine the effects of anxious symptomatology have generated conflicting data (Tully et al., 2016; Karlsen et al., 2021). In a 2014 study, Langvik and Nordahl (2014) adjusted for the effect of depression and confirmed that longitudinally, anxious symptomatology protects against acute MI. In a cross-sectional study by Huang et al. (2009) in a Taiwanese population, subjects with an anxiety disorder, but no depression, more frequently had coronary heart disease or hypertension as a comorbidity than healthy controls. Further, studies that have examined symptomatology by distinguishing between various presentations of anxiety disorders have noted an increased risk of CVD in panic disorder but not generalized anxiety disorder (McKellar et al., 2004; Tully et al., 2016). Although there is continued debate and heterogeneity in the literature regarding the separate effects of anxiety and depression and the precise mechanisms that are involved, these findings underscore the need for comprehensive care that includes the management of psychological distress, alongside traditional cardiovascular interventions.

On the behavioral level, depressed patients are two to four times less likely to adhere to their medication regimen (Romanelli et al., 2002), follow lifestyle recommendations (e.g., low-fat diet and smoking cessation) (Bradley and Rumsfeld, 2015) and practice self-management (McKellar et al., 2004; Benyamini et al., 2013; DiMatteo et al., 2000), whereas patterns of medical compliance in anxious patients have not been determined conclusively (Ai et al., 2012). Physical inactivity is also common among individuals with depression and anxiety disorders, which May contribute significantly to negative cardiovascular outcomes (Romanelli et al., 2002; Bradley and Rumsfeld, 2015). Depressive symptoms significantly impact LOS in cardiovascular patients, a relationship that has been highlighted by several studies. One key indicator of this influence is an elevated concentration of interleukin-6 in patients after surgery—this pro-inflammatory cytokine is linked to heart disease and psychiatric disorders, including depression (Ai et al., 2012; Kidd et al., 2014). These data implicate a connection between physiological and psychological factors in cardiovascular patients.

### 3.5 Stress and psychobiological responses: impacts on cardiac function

The relationship between psychological stress and cardiovascular health has been studied increasingly over the past two decades. When faced with a stressful situation, the body releases three main hormones to prepare an individual for an attack or escape response: adrenaline, norepinephrine and cortisol. During the former, adrenaline accelerates cardiac activity and respiration and increases blood pressure. Norepinephrine regulates the physical arousal that is needed to cope with the stressful situation. Finally, cortisol acts on the level of blood and muscle through the release of glucose and fatty acids to provide timely and sufficient energy to the body. Whereas hormone levels decrease once the stressful condition ceases under normal conditions, during chronic stress, these levels remain elevated, leading to increased cholesterol, glucose intolerance and hypertension—all elements that characterize the metabolic syndrome (Sara et al., 2018).

The literature (Poole et al., 2017) recognizes psychological stress as arising from appraisal processes, whereby an individual ascribes a certain meaning to events and various contexts into which he or she is placed, particularly to stimuli that are reputed to be threatening. Several brain systems are involved in such appraisal processes, which regulate peripheral physiological reactions to stress through visceral motor (brain–body) and visceral sensory (body–brain) mechanisms. A greater understanding of how the brain links stressful experiences to bodily changes would be useful to recognize and implement risk biomarkers to prevent and treat CVD.

Physiologically, the impact of stressful experiences is significant in the potential development of CVD. Brain imaging studies that have assessed the risk of CVD in relation to stress-induced cardiovascular reactivity suggest that specific brain areas—i.e., those that control central autonomic and physiological responses (cortical, limbic and brainstem)—are crucial in regulating stress-induced cardiovascular reactivity through the activation of visceromotor and viscerosensory mechanisms. Moreover, these areas mediate the cardiovascular reactions that are aroused by extreme stress or metabolic dysregulation; if they persist and intensify, these types of reactions can lead to various cardiovascular problems (Ginty et al., 2017). Thus, under conditions of vulnerability, dysregulation at the visceromotor and viscerosensory levels during stressful experiences can represent a cardiovascular risk factor (Poole et al., 2017).

Several studies (Martens et al., 2010; Kamardeen, 2021) have shown that psychological stress that is related to negative emotions negatively affects the degree of vulnerability to atherosclerotic CVD and constitutes a risk factor that equates to smoking, physical inactivity and dysregulated eating behaviors (Aw et al., 2023). A report on the correlation between perceived stress and incidence of CVD (AbuRuz et al., 2021) found that high levels of perceived stress are associated with a 27% rise in the risk of CHD, equivalent to consuming 5 additional cigarettes per day. The multidimensional nature of the relationship between perceived stress and adverse cardiovascular outcomes is also acknowledged, leading to the proposed existence of other intervening variables, such as greater activity of the hypothalamic–pituitary axis (Wu and Kling, 2016), increased sympathetic outflow (Horne et al., 2013) and altered behavioral patterns that cause insulin resistance and obesity (Gianaros and Jennings, 2018).

Despite the significance of this association, the exact mechanism that drives the link between stress and CVD remains unknown (AbuRuz et al., 2021). Many studies (Dimsdale, 2008; Rozanski, 2014; Richardson et al., 2012; Tsigos and Chrousos, 2002; Fredrikson and Matthews, 1990; Räikkönen et al., 1996; Ramadan et al., 2013) have identified mental stress as the origin of the physiological changes that significantly affect the incidence and development of heart disease. Specifically, psychological stress correlates with altered endothelial function, exaggerated peripheral microvascular tone and vasoconstriction of normal coronary segments. These vasomotor effects likely occur through the activation of stress response systems, causing coronary vasoconstriction and increasing heart rate and blood pressure, resulting in an imbalance in myocardial oxygen supply and demand.

Among its many physiological consequences, the literature has linked childhood maltreatment and elevated cardiovascular reactivity in clinical samples (Krantz and Burg, 2014) and has reported hypercortisolism in children with childhood maltreatment experiences (Hassan et al., 2009). Childhood exposure to ACEs, in fact, May disrupt physiological regulation in response to stress, particularly with regard to increased heart rate (HR) and blood pressure (Dakak et al., 1995). Consistent with this finding, excessive cardiovascular reactivity has been associated with atherosclerosis (Sherwood et al., 1999) and hypertension (Arrighi et al., 2000).

Similarly, prolonged and chronic exposure to ACEs can result in hyper-reactivity or hypo-reactivity of the autonomic nervous system and HPA axis (Soufer et al., 2009; Rinnewitz et al., 2018). HPA reactivity also correlates with coronary artery calcification and increased carotid IMT (Holochwost et al., 2020).

The polyvagal theory (Al'Absi et al., 2021; Roemmich et al., 2011) focuses on the role of vagal regulation in the autonomic nervous system’s response to changing environmental demands. Vagal regulation is related specifically to the function of the vagus nerve, the tenth cranial nerve, which is critical in transmitting parasympathetic influences to the heart (Chida and Steptoe, 2010). When an individual is at rest, the parasympathetic nervous system exerts inhibitory control over the heart, reducing its heart rate. This regulatory mechanism helps the body adapt to various situations and maintain a physiological balance in response to environmental changes (Del Giudice et al., 2011).

Measures of resting vagal activity provide insights into the body’s capacity to maintain a state of balance (homeostasis) and a general level of responsiveness during periods of relative stillness or inactivity. In contrast, vagal reactivity can be used to assess the body’s ability to respond and adapt to environmental demands and changes in circumstances (Carroll et al., 2012). Heart rate variability is a valuable non-invasive indicator of parasympathetic tone that helps capture the inhibitory influence of the vagus nerve on the heart’s sinoatrial node. Essentially, it reflects subtle fluctuations in the intervals between heartbeats, which can provide information about the state of the parasympathetic nervous system and its regulation of heart rate in response to various situations and stressors (Turner et al., 2020).

The recent literature has emphasized the relationship between vagal function (and autonomic imbalance) and CVD (Porges, 2003). Higher levels of vagal activity at rest and moderate levels of withdrawal on response are considered adaptive and effective regulatory processes (Porges, 2007). Low heart rate variability is associated with the risk of cardiovascular disorders, whereas increased heart rate variability is a protective factor during adaptation by the body (Porges, 2003).

Several meta-analyzes (Porges, 1995; Young et al., 2022) have examined vagal function and childhood adversity. Whereas certain studies (Wesarg et al., 2022) have not found an association between these variables, others (Sbarra and Borelli, 2013) have detected alterations in vagal regulation in clinical samples, indicating a relationship between resting-state vagal activity and maltreatment (Jarczok et al., 2019; Scrimin et al., 2018; Lavi et al., 2019; Sigrist et al., 2021).

## 4 Discussion

The cardiovascular risk of psychological factors has been studied for several decades, and the literature has highlighted the significant role of ACEs in cardiovascular disorders. Specifically, domestic abuse, physical abuse and sexual abuse have been identified as significant risk factors for the development of CVD and increased mortality.

This review has put in evidence some ACEs direct and indirect effects on late adult life through, for example, risky behaviors such as smoking and alcohol intake, which are generally recognized as independent risk factors for CVD. Gender also appears to influence the relationship between ACEs and CVD. Despite the conflicting findings, it appears that overall, women are more susceptible to the negative effects of ACEs on CVD, especially when they have experienced physical and sexual abuse. Further, female gender is associated with TTS, on which emotionally stressful events have a significant impact.

Based on our literature search, an insecure attachment style is emerging as a risk factor for the onset of heart disease, especially when associated with experiences of physical and sexual abuse, although there is no widespread consensus regarding the various types of insecure attachment. However, this evidence is derived from a small number of studies, which report that 70% of patients with congenital heart disease have experienced ACEs. The direct effect of these experiences on cardiovascular health should be examined further.

Attachment style represents a set of strategies for coping with fear. Consistent with studies on insecure attachment as a risk factor, this review has found that similarly, poorly adaptive coping strategies constitute a risk factor for the onset of CVD. These data suggest that analysis of attachment patterns by clinicians could benefit the care and management of patients with heart disease and promote better compliance with the care team.

Studies on coping styles have shown that the use of adaptive coping strategies, related to problem solving, is a protective factor that improves cardiovascular and inflammatory parameters. In contrast, maladaptive coping strategies, centered on managing negative emotions and associated with an insecure attachment style, heighten cardiovascular risk. The application of maladaptive coping styles, in fact, increases perceived stress, one of the most frequently studied parameters of cardiovascular risk. Highly stressful events, such as single-incident or chronic trauma (e.g., TTS and bereavement), worsen quality of life on the physical and psychological levels in patients with cardiovascular pathologies. Chronic stress also exacerbates the prognosis, independently or in combination with other factors. This finding underscores the weight of emotional stress as a factor to be considered in the various stages of the disorder.

In the complex relationship between psychological stress and cardiovascular disorders, in-depth psychological factors often coexist, necessitating the delineation of a patient profile that must be treated in a person-centered care setting.

All of these conditions are associated with a greater incidence of anxious and depressive symptoms, the indicators that are most frequently studied and found to be related to CVD. There is broad consensus that depression is a cardiovascular risk factor and is associated with an unfavorable prognostic outcome. In addition, depression is related to female gender, rendering women more vulnerable to early trauma outcomes. Although heart diseases tend to affect more men, it is possible that psychological risk factors have a greater impact on females. The factors above seem to have an impact on the recovery from cardiac events and to influence the length of hospital stay after surgery.

Of the medical and psychological aspects that have been discussed, the anxious attachment style was found to be an independent predictor of worse recovery. Further, post-traumatic stress encourages the use of avoidance strategies, which are useful in the short term but detrimental in the medium and long term.

Anxiety-depressive symptoms also have a negative impact on recovery and LOS. Identifying these indicators in the patient’s history and managing them during the hospital stay would allow a care team to improve the quality of care and reduce hospitalization time.

Based on our search and analysis of the literature, poor psychological health significantly impacts pathogenic pathways, the onset and course of CVD and recovery after the cardiac event. To this end, knowledge of the CVD and the psychological factors that are involved would make it possible to support and improve a patient’s health and quality of life, in the short and long term, and facilitate the development of integrated protocols for managing CVD patients. Future research should focus on examining the dual role of psychological factors in the emergence and progression of CVD.

The mixed results suggest that the relationship between ACEs, gender and CVD is complex and can vary, depending on individual factors and contexts. Additional studies are needed to fully understand the nuances of this relationship. There are few studies that have considered the role of attachment style in CVD patients, how this relates to coping strategies and, consequently, to the patient’s perception of hospitalization.

The evidence recognizes the importance of anxiety-depressive symptoms in CVD patients. However, there is a lack of studies on the impact of symptomatology that have isolated one condition from another to assess the specific effects of each.

## 5 Limitations and future research

This study has several methodological limitations that reduce the generalisability of the results. In particular, the narrative nature of the review and the absence of a systematic analysis limit the ability to identify and critically evaluate all available evidence on the topic. Furthermore, the lack of differentiation between different cardiovascular diseases prevents a deeper understanding of the specific associations between psychological variables (such as stress, psychological disorders, personality factors, and coping strategies) and various clinical manifestations.

Finally, the retrospective assessment of early stress introduces a further limitation, as under- or over-estimates of the impact of early stress on cardiovascular health May occur by means of self-report instruments. In fact, retrospective assessment of stress is subject to memory bias and the tendency of individuals to re-evaluate past events in light of current experiences.

Future research, particularly on the impact of early stress on cardiovascular health, should include more prospective and longitudinal studies, which require significant resources. Although the literature review has revealed a picture in which the factors examined are interrelated, there are critical issues related to methodological structure or simple lack of studies, which remain gaps in our understanding of these relationships.

Additional studies could clarify the relationship between ACE, gender and CVD, taking into account individual factors and contexts. It would also be interesting to investigate the role of attachment style in patients with CVD, in order to define its influence on coping strategies and perceptions of hospitalization. Starting from the well stated importance of anxious-depressive symptoms in patients with CVD, it would be useful to further explore the impact that the two symptomatic frameworks might have independently of each other.

All in all, this study highlights the importance of treating psychological distress in patients with CVD. In this regard, recent studies suggest available interventions to address anxiety and emotional issues, such as imagery techniques (Sebri et al., 2024), or other integrative approaches for stress management (Carlson et al., 2019; Nakao et al., 2021; Jerath et al., 2023).

## 6 Conclusion

Based on this literature review, the psychological factors that are connected to cardiovascular pathologies have been studied, on various levels, for over 40 years. That the literature as a whole has demonstrated how psychological factors contribute to the emergence of cardiovascular pathology and then significantly impact the course of these pathologies leads us to conclude that psychological variables and cardiac health must be considered in an integrated manner. Our review aims to inspire global patient care—not only to improve the quality of care but also to achieve a better understanding of the mechanisms that underlie the various pathologies and structure more effective prevention and intervention programs in terms of cost and efficacy.
